# High uterosacral ligament hysteropexy for the management of pelvic organ prolapse

**DOI:** 10.1590/S1677-5538.IBJU.2020.0384

**Published:** 2021-03-10

**Authors:** Naveen Kachroo, Samantha Raffee, Solafa Elshatanoufy, Humphrey Atiemo

**Affiliations:** 1 Vattikuti Urology Institute Henry Ford Hospital DetroitMI USA Vattikuti Urology Institute, Henry Ford Hospital, Detroit, MI, USA.; 2 Glickman Urological and Kidney Institute Cleveland Clinic ClevelandOH USA Glickman Urological and Kidney Institute, Cleveland Clinic, Cleveland, OH, USA.

## Abstract

**Objective:**

To demonstrate our transvaginal high uterosacral ligament (HUL) hysteropexy technique as an alternative mesh-free uterine-preserving pelvic organ prolapse (POP) repair approach and present our institutional outcomes. Concurrent hysterectomy with POP repair is controversial as uterine-preserving techniques may beneficially allow fertility, body image and sexual function preservation (1, 2).

**Materials and Methods:**

This video illustrates a step-by-step sequence of our HUL hysteropexy technique in a symptomatic Stage III POP patient. Retrospective single-institution, single-surgeon analysis of patients treated by either HUL hysteropexy or hysterectomy with HUL suspension for symptomatic prolapse was performed with minimum 2 years of follow-up. Patient demographics, operative characteristics, pre and post-operative POP-Q evaluation, American Urological Association Symptom scores (AUASS) and post-operative Pelvic Floor Distress Inventory (PFDI-20) were compared.

**Results:**

Surgery time was 3 hours 24 minutes. No immediate/early complications were noted, with successful repair on follow-up. Outcomes of 18 patients (10 HUL hysteropexy, 8 hysterectomy and HUL suspension) were assessed (Supplemental Table). The only baseline difference was a lower body mass index in the HUL hysteropexy cohort (25.8 vs. 35.8kg/m2, p=0.008). In the HUL hysteropexy cohort, blood loss (mean: 58 vs. 205ml, p=0.00086) and operative time (190.4 vs. 279.1minutes, p=0.0021) were significantly reduced. There was no difference in post-operative AUASS, POP-Q or PFDI-20 at 2 years.

**Conclusion:**

We present our HUL hysteropexy technique. Although limited by sample size and retrospective design, resulted in significantly reduced blood loss and operative time with comparable post-operative 2 year outcomes to non-uterine-preserving techniques. In our opinion, HUL hysteropexy is a safe, durable POP management option for women without significant endometrial pathology risk factors.

## SUPPLEMENTAL TABLE

**Table 1:** Comparison of outcomes of women undergoing high uterosacral ligament hysteropexy versus hysterectomy and high uterosacral ligament suspension for pelvic organ prolapse repair.

	High Uterosacral Ligament Hysteropexy	High Uterosacral Ligament Suspension with Hysterectomy	p-value
Number	10	8	
Mean Age (years)^a^	69.1±14.18	65±13.00	0.562
Mean BMI (kg/m^2^)*^a^	25.81±4.48	35.84±6.72	0.008
Median Parity	3	3	
Race	Caucasian: 11	Caucasian: 6	
	African American: 3	African American: 4
		Other: 3	
Preop POP-Q Stage	Stage 2: 30%	Stage 2: 25%	0.306
	Stage 3: 50%	Stage 3: 75%
	Stage 4: 20%	Stage 4: 0
AUASS Pre-op^a^	19.9±6.06	14.5±9.15	0.230
AUASS Post-op^a^	11.13±9.38	13.00±6.24	0.453
Operative Time (minutes)*^a^	190.40±41.89	279.13±39.01	0.0021
EBL (mL)*^a^	57.50±29.08	205.00±94.38	0.00086
Mean Follow-up (months)	33.25	36.71	0.410
Post-op PopQ stage (at 2 years in pts not requiring reoperation)	Stage 0: 0%	Stage 0: 0%	0.282
	Stage 1: 12.5%	Stage 1: 50%
	Stage 2: 87.5%	Stage 2: 50%
	Stage 3+4: 0%	Stage 3+4: 0%
Post-op PFDI-20 (at 2 years in pts not requiring reoperation)^a^	18.50±17.61	26.93±16.16	0.483

**BMI** = Body Mass Index;

**EBL** = Estimated Blood Loss;

**AUASS** = American Urological Association;

**POPQ** = Pelvic Organ Prolapse Quantification;

**PFDI** = Pelvic Floor Distress Inventory.

* statistically significant difference

a Mean +/- Standard Deviation
